# Electrically tuning soft membranes to both a higher and a lower transparency

**DOI:** 10.1038/s41598-019-56505-9

**Published:** 2019-12-27

**Authors:** Leihao Chen, Michele Ghilardi, James J. C. Busfield, Federico Carpi

**Affiliations:** 1 School of Engineering and Materials Science, Queen Mary University of London, London, E1 4NS United Kingdom; 2 Department of Industrial Engineering, University of Florence, Florence, 50139 Italy

**Keywords:** Mechanical engineering, Polymers

## Abstract

The possibility to electrically tune the optical transparency of thin membranes is of significant interest for a number of possible applications, such as controllable light diffusers and smart windows, both for residential and mobile use. As a difference from state-of-the-art approaches, where with an applied voltage the transparency can only increase or decrease, this paper presents the first concept to make it electrically tuneable to both higher and lower values, within the same device. The concept is applicable to any soft insulating membrane, by coating both of its surfaces with a circular transparent stretchable conductor, surrounded by a stretchable annular conductor. The two conductors are used as independently addressable electrodes to generate a dielectric elastomer-based actuation of the membrane, so as to electrically control its surface topography. We show that the optical transmittance can electrically be modulated within a broad range, between 25% and 83%. This approach could be especially advantageous for systems that require such a broad tuning range within structures that have to be thin, light**w**eight and acoustically silent in operation.

## Introduction

Smart membranes with optical transparency that can be electrically tuned are gathering growing interest for possible applications in various fields. For instance, controllable light diffusing filters that can continuously adjust the degree of light diffusion are used to either avoid eye damage from direct spot lights, hide details or obtain soft illumination^1^. As another example, so-called smart windows are being employed to replace conventional sunlight shades, for instance in new-generation passenger airplanes^2^. Similarly, smart privacy glass is being used as a substitute for louver blinds in modern buildings^3^. At present, the majority of such devices with electrically controllable transparency is based on electrochromic materials, where an electric field-driven transport of ions between an ion storage film and an electrochromic film is used to modulate the optical transmittance^3–6^. Other types of devices are based on polymer-dispersed liquid crystals (PDLC), where the application of a voltage changes the alignment of liquid crystal molecules, so as to electrically switch the transparency^7–10^.

**Figure 1:**
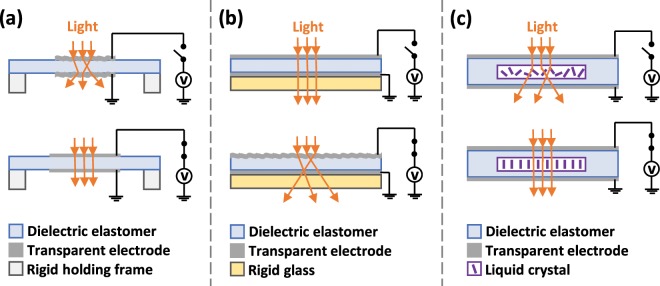
State-of-the-art strategies to achieve DEA-based devices with electrically tuneable optical transparency: (**a**) expansion of a soft membrane with wrinkled stretchable transparent electrodes; (**b**) cratering-type surface instability of a constrained soft membrane with transparent electrodes; (**c**) alignment of liquid crystals encapsulated within a soft matrix sandwiched between transparent electrodes. With strategy (**a**), at el**e**ctrical rest the wrinkled electrodes cause extensive scattering of the transmitted light, whilst a voltage-induced surface expansion flattens the wrinkles, thereby increasing the transparency. With strategy (**b**), the tr**a**nsparency of the device at electrical rest is reduced by an applied voltage, due to electromechanical instabilities that form craters, which scatter light. With strategy (**c**), at electrical rest the twisted liquid crystals scatter light, whilst voltage-induced alignments of the crystals increase the transparency. Note: the change of membrane thickness resulting from the actuation of each device is not shown for simplicity.

Recently, so-called dielectric elastomer actuators (DEAs) have been shown as an alternative promising technology to achieve large and continuous voltage-induced changes in optical transparency^11–18^. DEAs, which belong to the broader family of electromechanically active polymers^19^, essentially are electrically deformable capacitors, typically consisting of a dielectric elastomer membrane carrying two compliant electrodes. By applying an electric field across the membrane, the structure can continuously and reversibly deform, showing surface expansion and thickness compression^20,21^. The deformation is induced by an effective electrostatic pressure *p* = *ε*_0_*ε*_r_*E*^2^, where *ε*_0_ is the dielectric permittivity of vacuum, *ε*_*r*_ is the material’s relative dielectric constant and *E* is the applied electric field^21^. The DEA technology is in general suited to obtain electrically adaptive devices that exhibit large strains, fast response, high resilience, lightweight, high energy efficiency and no acoustic noise^22–24^. In recent years, a diversity of DEA-based devices has been reported for optical applications, including electrically tuneable lenses, diffraction gratings, phase retarders and colour changing devices^25–30^. DEAs have also been used to electrically tune the optical transparency of soft insulating membranes, with various strategies, which can be grouped as presented in Fig. 1.

The first strategy (Fig. 1a) consists in coating the soft dielectric membrane with stretchable transparent electrodes, which are initially made translucent by mechanically inducing an electrode wrinkling and then are made more transparent by electrically inducing an electrode smoothing^11–13^. In a second approach (Fig. 1b), the transparency can be reduced by means of cratering-type surface electromechanical instabilities of transparent electrodes covering the membrane fixed to a rigid substrate, typically requiring very high electric fields^14–17^. In a third approach (Fig. 1c), voltage-controlled light shutters are obtained by encapsulating liquid crystals into the membrane and sandwiching it between transparent electrodes, to achieve an operation similar to that of PDLC windows, producing on-off switching^18^. According to all these state-of-the-art approaches, by using electrical means the optical transparency can only be increased or decreased.

Here, we present the first concept to make the optical transparency electrically tuneable to both higher and lower values, within the same device. The concept is applicable to any soft insulating membrane, by coating both of its surfaces with a central circle of transparent stretchable conductor, surrounded by a stretchable conducting ring. We show how the resulting transparency of the central area can be electrically modulated with dual operation, such that it can be both increased and decreased, depending on whether the electrical activation is used to generate an expansion or a contraction.

## Results and Discussion

### Electrically induced expansions and contractions of a soft membrane to tune its transparency

**Figure 2:**
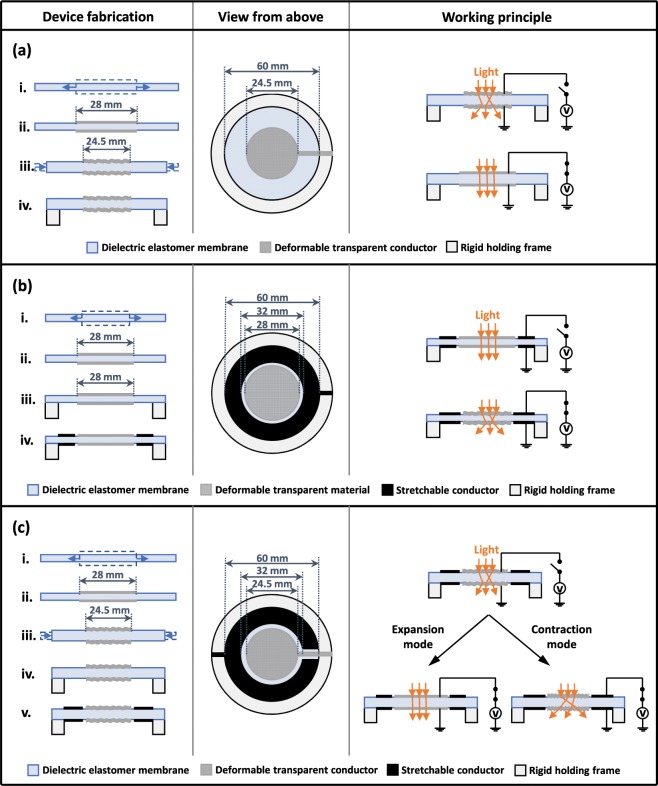
Concepts for a DEA-based electrical tuning of the optical transparency of a soft membrane working in (**a**) expansion mode, (**b**) contraction mode or (**c**) dual expansion-contraction mode. Note: the (non-uniform) change of membrane thickness resulting from the actuation of each device is not shown for simplicity.

The dual-operation concept proposed in this work originates from the combination of the expansion-mode operation mentioned above (Fig. 1a) with a contraction-mode operation, based on a different structure described below. The two operation modes and their integration into a dual-mode single device that can exploit both of them are schematically presented in Fig. 2.

The expansion-mode device (Fig. 2a) is obtained as follows. The elastomeric membrane is firstly biaxially pre-stretched and then coated on both sides with a transparent deformable conductor to create electrodes, which define a circular DEA. By making the electrode’s stiffness higher than that of the elastomer, it is possible to create wrinkles on the electrode layer when the pre-stretch is reduced. After that, the membrane is clamped to a rigid circular frame. At electrical rest, the wrinkled electrodes scatter the transmitted light, making the sandwich opaque. When a voltage is applied, the resulting surface expansion causes the wrinkles to flatten, thereby reducing the scattering and so leading to a higher optical transparency (Fig. 2a). Therefore, this configuration, known from the state of the art, translates increasing voltages into an increasing transparency.

On the other hand, if any given application requires that the membrane at electrical rest is transparent and then should become progressively opaque with increasing voltages, a different concept is required. To achieve this, in this work we envisaged a contraction-mode device presented in Fig. 2b. It is obtained by firstly biaxially pre-stretching the elastomeric membrane and coating both sides of it with a transparent deformable material on a central area. The membrane is then fixed to a rigid holding frame and a conductive stretchable material is applied, on both sides, creating an outer ring, separated from the central circle. That ring (which can be non-transparent) works as the stretchable electrode of an annular DEA, whilst the central circle (which can be non-conductive) acts as a deformable medium with strain-dependent scattering of the transmitted light. When the device is at electrical rest, the central coating is optically transparent. When a voltage is applied, the annular electrodes expand towards the centre (as they are constrained at their outer edge), causing the central area of the membrane to relax, due to a reduction of its pre-stretch. If the stiffness of the central coating is higher than that of the elastomer, the resulting effect is the creation of wrinkles, which scatter light and so reduce the transparency (Fig. 2b). Therefore, this alternative configuration allows for using increasing voltages to decrease the transparency.

Nevertheless, there might be application scenarios where it could be of interest to electrically tune the transparency to both higher and lower values, within the same membrane. To address this need, we conceived an integration of the two concepts described above, leading to the dual-mode (expansion-contraction) device shown in Fig. 2c. It can be assembled using the same procedure adopted for the expansion-mode device (Fig. 2a), although in this case both the surfaces also host outer ring-shaped stretchable electrodes, to form an annular DEA, as for the contraction-mode device (Fig. 2b). Note that the central transparent material is not only deformable, as required by the contraction mode, but also electrically conductive, as required by the expansion mode. On each surface, the outer ring electrode encircles the central circular electrode without contact between them, so that the two can be electrically activated independently. Indeed, their independent driving enables a dual operation of the device: the transmitted light scattering caused by the wrinkled central area at electrical rest can be either increased or decreased, depending on whether the electrical activation concerns the annular DEA (contraction mode) or the circular DEA (expansion mode), respectively (Fig. 2c).

The three concepts described above were implemented to investigate how the proposed dual-mode configuration performs as compared to the single-mode ones. The experiments were based on pre-stretched dielectric elastomer membranes made of a commercial acrylic elastomer film (VHB tape series, by 3M), as detailed in Methods. This elastomer was chosen because of its extensive usage within the DEA field as a test material, due to a high electromechanical transduction performance in quasi-static conditions and a simplification of manufacturing processes resulting from its adhesive properties^20–24^.

The most challenging aspect of the implementation was the material to be used as transparent deformable conductor. The state of the art offers a diversity of materials^31^, including silver nanowires^14^, metal oxide thin films^11,12^, carbon nanotubes^17,32^, graphene^33^, ionogels^34^ and conducting polymers^35^. Among them, PEDOT:PSS was selected as a conducting polymer extensively used for transparent electrodes in flexible organic electronic devices^36^. PEDOT:PSS can offer transparency and conductivity comparable to those of indium-tin oxide electrodes^37,38^. Nevertheless, the order of magnitude of its Young’s modulus in pure form is usually as high as 1 GPa^39^. This would prevent PEDOT:PSS from serving as an effective compliant electrode for DE actuation. However, the addition of dimethylsulfoxide (DMSO) and a zonyl fluorosurfactant into an aqueous PEDOT:PSS solution makes it possible to produce transparent PEDOT:PSS electrodes that are stretchable^40,41^, with reported retentions of conductivity for strains in excess to 180%^36^. Therefore, in this work the membrane was spray coated with a thin layer of PEDOT:PSS to create the central stretchable transparent conducting area, and for the contraction- and dual-mode devices a carbon grease was used to create the outer non-transparent stretchable electrodes. Details about materials and fabrication processes are reported in Methods.

These materials were used to assemble the three devices described above. In order to obtain a roughly symmetrical behaviour and enable proper comparisons of their performance, they were carefully sized as follows. They all had the same membrane’s outer diameter, so as to have the same size constraint. With respect to the reference configuration represented by the expansion-mode device, the PEDOT:PSS coated area of the contraction-mode device had at electrical rest the same diameter (28 mm) that the expansion-mode device had prior to wrinkling during manufacturing (Fig. 2). With this choice, the contraction-mode device had at electrical rest the same transparency and same window size of the expansion-mode device when its wrinkles are electrically flattened; moreover, with an applied electric field sufficiently high to reduce the diameter to the same value that the reference device has at rest (24.5 mm), the contraction-mode device was made able to reach the same opacity (wrinkling) of the reference device at rest (Fig. 2). This behaviour was confirmed by the tests presented in the next section. For the dual-mode device, the PEDOT:PSS diameter was the same as that for the reference device (24.5 mm), so as to achieve the same effect when used in the expansion mode (Fig. 2).

The size of the annular electrode in the contraction- and dual-mode devices was identical, in order to ensure an analogous effect. Indeed, for any given active strain of the annular surface, a wider annulus would have a larger deformation, inducing a higher strain of the central region. In particular, the annulus width (difference between its outer and inner diameters) was made identical (14 mm) to the radius of the PEDOT:PSS coated area of the expansion-mode device (Fig. 2).

This sizing of the three devices enabled a comparative investigation of their performance, which is presented below.

### Electrical tuneability of the soft membrane transparency

The electro-optical transduction performance of each device was characterised in the 400–800 nm visible range (see Methods), quantifying how different electrical activations changed the optical transmittance measured in both the near and far fields. According to the test equipment used in this work, near and far fields are here defined as a device-to-detector distance of 5 and 40 cm, respectively (see Supplementary Fig. S1).

In the near field, the measurements concerned both the diffuse transmittance *T*_*d*_ (percentage of incident light transmitted as diffused light) and the total transmittance *T*_*t*_ (percentage of incident light transmitted as a whole, including both diffused transmitted and specular transmitted components, i.e. scattered and not), which were detected as detailed in the Supplementary Fig. S1. The data were also used to calculate the transmission Haze, defined as the ratio between the near-field diffuse and total transmittances: *Haze* = *T*_*d*_/*T*_*t*_. The Haze value, representing the percentage of total transmitted light that propagates as diffused light, was used to give an indication of the cloudy appearance of the sample^42^.

In the far field, we simply considered a far-field transmittance, without any further distinction between diffused and specular components, due to the fact that such a difference tends to progressively lose significance as the sample-to-detector distance increases (the diffused component tends to be lost by the measurement equipment - see Supplementary Fig. S1).

**Figure 3:**
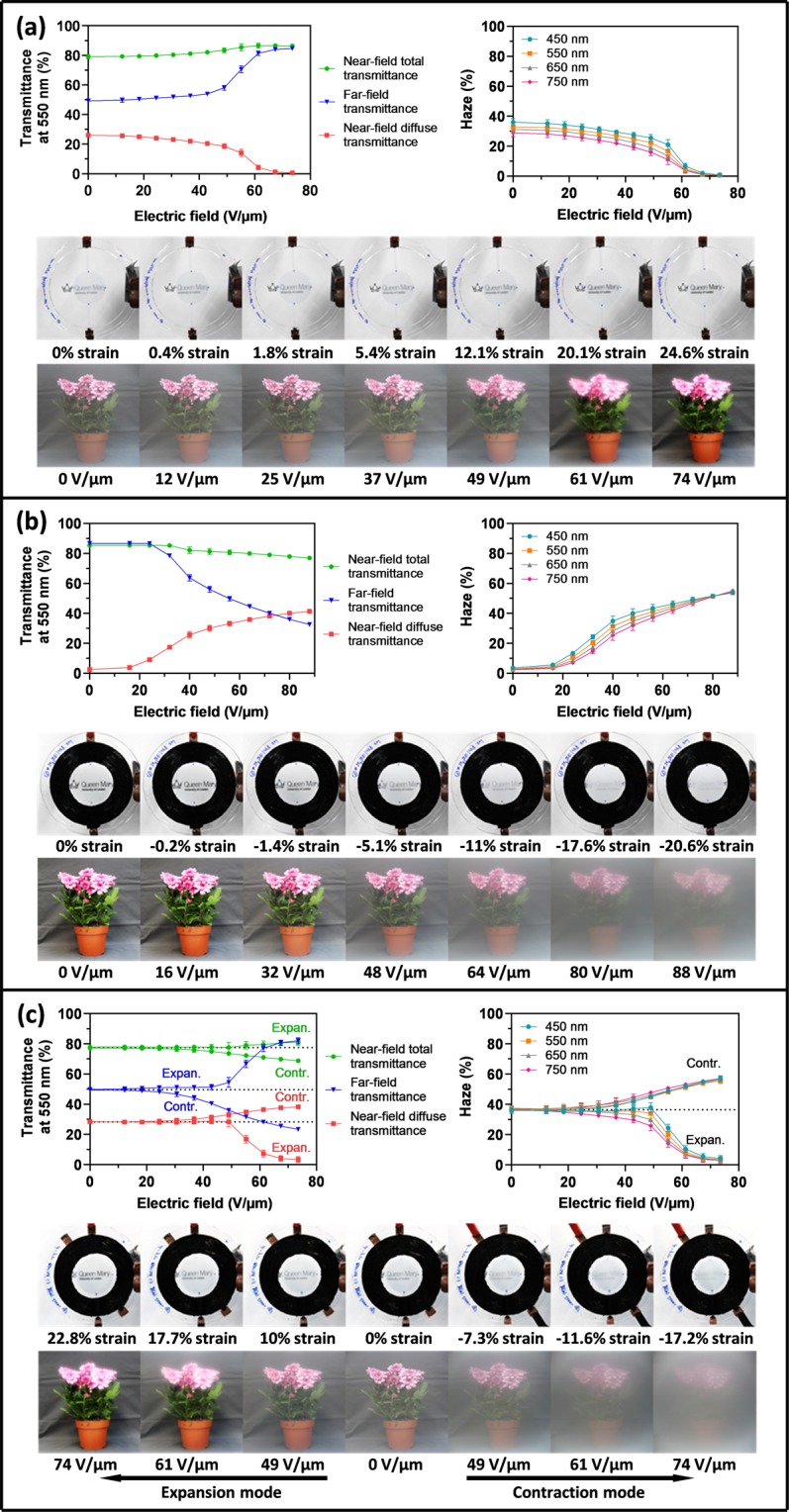
Electro-optical transduction performance of the tested soft membrane-based devices working in (**a**) expansion mode, (**b)** contraction mode or (**c**) dual expansion-contraction mode. For each configuration, the electrically-induced variations of the transmittances (near-field total, near-field diffuse and far-field) at 550 nm and the Haze number at different wavelengths are shown. Each data point represents the average value from three sample devices. Error bars corresponding to the standard deviation are included, although most of them are too small to be seen within the 0–100% range of the graph. The photographs visualise the change in transparency due to the specified electric fields, which caused the reported area strains of the PEDOT:PSS window: the first photo set shows the device covering text 3 cm away, whereas the second set shows flowers approximately 100 cm away from a device attached to the camera lens.

The near-field total and far-field transmittances of the pre-stretched acrylic elastomer membranes (without any coating) were approximately 92% at 550 nm, whilst their Haze value was lower than 0.5% at 550 nm. The electro-optical transduction performance achieved while using the membranes in the three described configurations to tune their transparency is presented in Fig. 3, whereas their corresponding optical spectra are given in the Supplementary Fig. S2.

Owing to the transparency of PEDOT:PSS thin film, all the devices had at electrical rest a rather high near-field total transmittance (around 80%, Fig. 3), which could then be electrically increased or decreased, according to either the expansion or contraction mode, respectively. Any electrically-induced increase/decrease of the near-field total transmittance (green curves in Fig. 3) was due to a smoothening/wrinkling of the surface, which caused a reduction/increase of the near-field diffuse transmittance (red curves in Fig. 3) and an increase/decrease of the far-field transmittance (blue curves in Fig. 3).

Interestingly, in all the cases the near-field total transmittance corresponded rather accurately (±5% error) to the sum of the near-field diffuse transmittance and the far-field one (Fig. 3). This means that the sample-to-detector distance adopted in the far field (40 cm) was sufficiently long that in practice nearly all the diffuse component of the transmitted light was not detected (Supplementary Fig. S1), such that the far-field transmittance was essentially representative only of light that was specular transmitted, i.e. not scattered. The so-achieved far-field transmittance then provides a quantification of the transparency that an observer can perceive while looking through the device window, perpendicularly to it, from that distance.

From a qualitative standpoint, the electrical tuneability of the transparency can be observed from the photos in Fig. 3, which were taken by arranging the devices either in front of a camera (to image the whole device and a text behind it, also showing the electrode deformation at different electric fields) or attached to its lens (to image a whole scene with flowers as seen through the device window). The controllability of the dual-mode device is also visible in the Supplementary Video SV1. The cloudy appearance of the three devices, as a result of their electrically variable scattering of light, is quantified by the Haze-field plots (Fig. 3).

**Figure 4:**
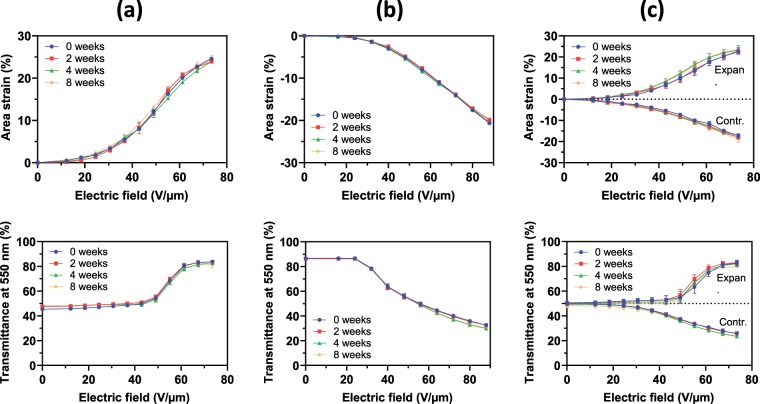
Electro-mechano-optical performance stability over time. The dependence on the applied electric field of both the area strain of the PEDOT:PSS-coated circle and its far-field transmittance at 550 nm are shown at different times after fabrication for devices operating in (**a**) expansion mode, (**b**) contraction mode or (**c**) dual expansion-contraction mode, respectively. Each data point represents the average value from three sample devices. Error bars corresponding to the standard deviation are included, although most of them are too small to be seen within the 0–100% range of the graph.

The electrically induced area strains experienced by the PEDOT:PSS coated circles are presented in Fig. 4. In particular, strain-field and transmittance-field plots are shown with data sets taken both immediately after fabrication and up to eight weeks later.

The first evidence is that the electro-mechano-optical performance was found to be particularly stable within the monitored time span. Moreover, the strain-field plots provide further insights on how the electrical actuation of the membrane in each configuration affects its transparency, as discussed below. For the sake of comparisons, in the following we consider data at a maximum applied field of 74 V/µm, as it is the highest available for all the three cases (Fig. 4)

For the expansion-mode device, the area strain at 74 V/µm (about 24.6%, Fig. 4a) corresponded to an increase of the PEDOT:PSS diameter from 24.5 to 27.4 mm, which was close to the size before the creation of the wrinkles during manufacturing (28 mm, Fig. 2a). Therefore, the electrical activation of the wrinkled electrodes could recover a nearly smooth surface. Moreover, as 28 mm was also the PEDOT:PSS diameter of the contraction-mode device at electrical rest (Fig. 2b), the transparency electrically reached by the expansion-mode device was basically the same as that of the contraction-mode device at rest. This was confirmed by the transmittance measurements (Fig. 4a,b).

For the contraction-mode device, the area strain at 74 V/µm (about −15%, Fig. 4b) corresponded to a reduction of the PEDOT:PSS diameter from 28 to 25.8 mm, which was close to the size of the expansion-mode device at electrical rest (24.5 mm, Fig. 2a). So, the electrical activation of the contraction-mode device was able to create a level of wrinkling comparable to that of the expansion-mode device at rest, and so also a comparable scattering of light. This was confirmed by the transmittance measurements (Fig. 4a,b).

For the dual-mode device, the operation in expansion or contraction mode created at 74 V/µm a strain of 23% or –17.2%, respectively (Fig. 4c), changing the PEDOT:PSS diameter from 24.5 mm to either 27.2 or 22.3 mm, respectively. Therefore, when used in expansion mode, the device behaved like the single expansion-mode one; this was confirmed by the transmittance measurements (Fig. 4a,c). When used in contraction mode, however, the lower strain achieved made the electrical reduction of the transmittance smaller than that occurring in the single contraction-mode device (Fig. 4b,c).

The creation and relaxation of surface wrinkles underpinning this electro-mechano-optical performance was studied with a microscopic investigation, described below.

### Microscopic investigations on the reversible surface wrinkling

**Figure 5:**
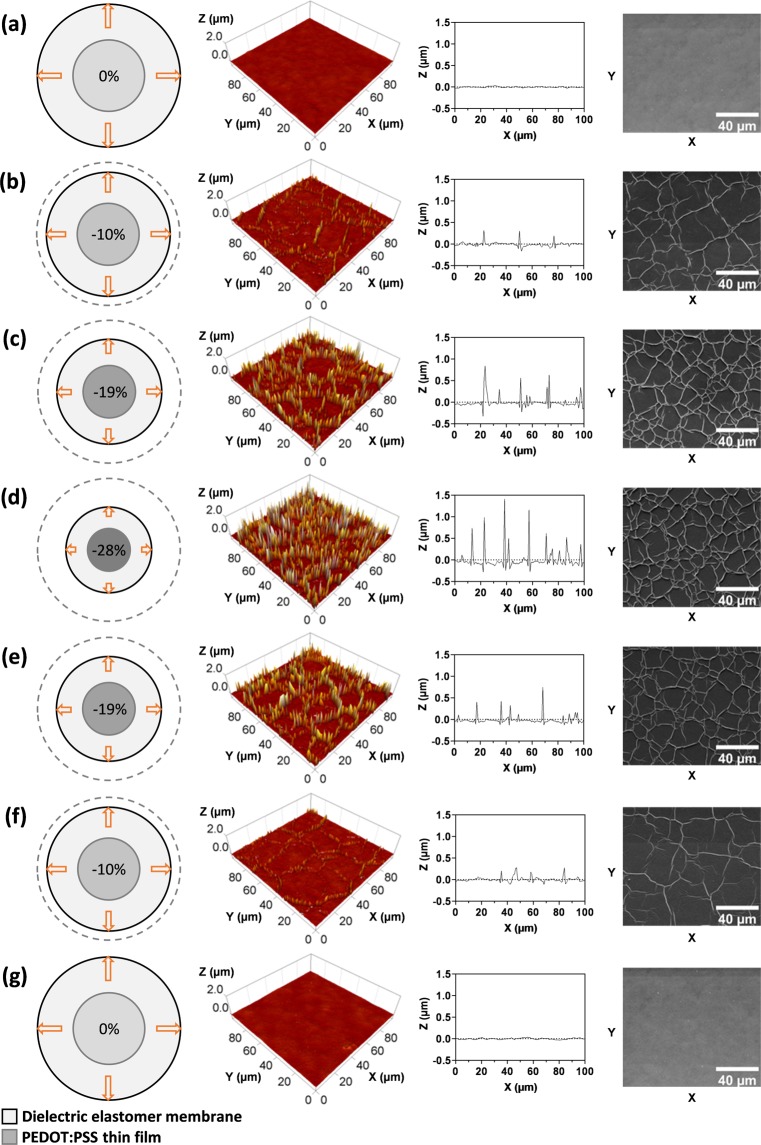
Investigation on the reversible surface wrinkling of the PEDOT:PSS thin film covering the elastomer membrane, as a result of different imposed strains. AFM plots (second and third columns) and SEM images (fourth column) of the surface are shown at an area strain of (**a**) 0%, (**b**) −10%, (**c**) −19% and (**d**) −28%, resulting from a progressive reduction of the membrane stretch, and then at an area strain of (**e**) −19%, (**f**) −10% and (**g**) 0%, resulting from a re-increase of the membrane stretch.

Atomic force microscopy (AFM) and scanning electron microscopy (SEM) were used to investigate the reversible formation of surface wrinkles within the PEDOT:PSS thin films at different area strains. The strain values were selected as representatives of those experienced by the PEDOT:PSS-coated region in the three devices, either mechanically or electrically. To this end, the acrylic elastomer membrane was first biaxially pre-stretched by 4 times and then coated with a thin circular layer of PEDOT:PSS. From this condition, the membrane was partially relaxed by reducing the stretch, such that the PEDOT:PSS circle was subject to area strains of −10%, −19% and −28%. Subsequently, the strains were reversed back to −19%, −10% and 0%, by progressively stretching the membrane again, so as to investigate the surface wrinkling reversibility. The results are presented in Fig. 5.

As revealed by both the AFM and SEM images, the initial smooth surface of the as-created PEDOT:PSS film (Fig. 5a) showed progressively growing wrinkling as the area progressively contracted (Fig. 5b-d). In particular, the wrinkles increased in number (surface density) and height, with peak values ranging from about 0.3 µm at a −10% strain to about 1.5 µm at a −28% strain (Fig. 5). These morphological surface changes were fully reversible, as shown by the images taken at strains that were progressively reverted back to 0% (Fig. 5e–g).

Therefore, as the electrical actuation of the membranes induced strains analogous to those used in these tests, these results provide evidence that the electrical tuning of the membrane transparency occurs via a surface wrinkling that shows up with topological patterns that can be controlled in a continuous and reversible manner.

### Performance comparisons

**Figure 6:**
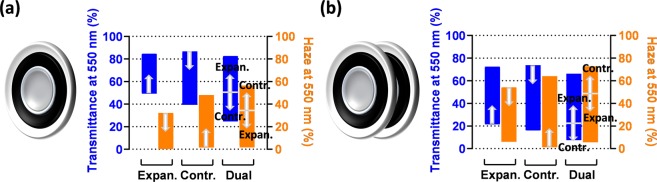
Tuning ranges of the far-field transmittance and Haze value at 550 nm for the three operating modes. Data are presented for (**a**) a single device and (**b**) two coaxially aligned devices. (**b**) The arrows indicate the directions of variation for increasing electric fields, up to 74 V/µm.

In order to compare how the three driving modes affect the electro-optical performance, Fig. 6a reports for the three cases the tuning ranges of the far-field transmittance and the Haze value (both at 550 nm), for electric fields up to 74 V/µm.

Compared to the expansion-mode configuration, the contraction-mode one offered a wider tuning range, both for the transmittance and the Haze value. With respect to the contraction-mode device, the dual-mode configuration was capable of a broader range, both for the transmittance and the Haze (Fig. 6a), although its main advantage clearly is the possibility to reach both higher and lower transparencies from the rest condition.

In order to increase the portion of scattered light and so increase the tuning range, the same investigations presented above were repeated on another set of three systems, each consisting of two coaxially-aligned, 3 mm-spaced devices, operating in parallel according to one of the three modes described above. Therefore, light was forced to pass through total four layers of wrinkled PEDOT:PSS films. The characterisation of these systems is presented in the Supplementary Figs. S3 and S4, whereas the resulting tuning ranges are summarised in Fig. 6b.

It is evident that, for each driving mode, the double-membrane system outperformed the single-membrane one in terms of tuning range amplitude (Fig. 6). However, the doubled number of wrinkled PEDOT:PSS layers reduced the maximum transmittance that can be achieved among the three devices, from more than 85% to about 75% (Fig. 6). Therefore, doubling the number of membranes/aligned devices is a useful strategy to increase the tuning range, although at the cost of a reduced maximum transparency.

## Conclusions

Three ways to electrically tune the optical transparency of soft elastomeric membranes, using dielectric elastomer actuation, were presented and compared. The driving strategies were based on different methods to electrically modulate the surface topography of a transparent PEDOT:PSS thin film covering the membrane surface.

The configurations operating in expansion/contraction modes were used to electrically increase/decrease the light transmittance, whilst the dual-mode configuration enabled a modulation of transparency to both higher and lower values, with the broadest tuning range of far-field transmittance (25%-83%). Within the monitored time span of 8 weeks, all the implemented devices exhibited stable electro-mechano-optical performance.

Coaxially aligning two of such devices served to broaden the transmittance tuning range (7%-66%), although at the cost of reducing the transmittance maximum value.

Future developments could improve the device performance by optimising the area ratio of the two independent electrodes, according to application-driven specifications in terms of required window size and acceptable device size. To this aim, a useful tool would be represented by computational models, such as that by Lu *et al*.^43^.

Improvements could also concern the use of conductive deformable transparent materials with higher refractive index, in order to increase the scattering of light when the surface is wrinkled, and so further extend the transmittance tuning range. The significant diversity of stretchable transparent materials available today, as recalled in the introduction, offers a wide range of choices to be explored for possible improvements.

Furthermore, the use of membranes made of silicone elastomers is expected to lead to devices capable of the highest possible tuning speeds, as it can be inferred from previous investigations on tuneable lenses driven by dielectric elastomer actuation^26^.

As an example of application, the various configurations described in this paper to electrically tune the transparency of elastomeric membranes could be useful for tuneable ‘windows’. They are here generically defined as any interface that separates distinct environments and is able to transmit light. So, they are not necessarily limited to conventional windows used in buildings and vehicles. Depending on the specific type of system/application of interest, and, so, the specific functionality required, a device configuration could be more advantageous than others. For instance, for a window of a building, the need is usually not to enable both increases and decreases of the transparency, but, rather, just to reduce it (e.g. during sunshine) from a high value at rest. So, in this case, the contraction-mode device would be more useful. Differently, there might be other systems/applications, such as windows acting as light filters in optical machines, where the need for commuting to both a lower and a higher transparency could justify the use of the dual-mode device, even at the cost of requiring a constant voltage to keep the maximum-transparency state.

Therefore, as for any other technology, the selection of a configuration for a given application should weight the pros and cons according to the functional needs. For that selection, moreover, the energy consumption requirements should be considered as well. With regard to this, it is worth stressing that driving any of the proposed configurations with a constant voltage would consume very limited current (just leakage current) and so, very limited power, due to the capacitive nature of the load (as for any DEA). This property, combined with the characteristic thin structure, low specific weight and acoustically silent operation, makes this technology attractive to electrically control the transparency of soft membranes.

## Methods

### Dielectric elastomer membrane

All the tests were performed using clear and adhesive acrylic-based elastomer membranes (VHB 4910, 3M, USA). The membranes were biaxially pre-stretched (see the values specified below), in order to achieve a well-known increase in the electromechanical transduction performance, as first documented by Pelrine *et al*.^20^ and later on explained in different ways by Brochu and Pei^22^ and Koh *et al*.^44^ The membrane had at rest a thickness of 1 mm, which then reduced to a lower value, depending on the applied pre-stretch, as detailed below.

### Stretchable transparent conductive material

Stretchable, conductive and transparent layers on the dielectric elastomer membranes were obtained by spray coating a PEDOT:PSS compound, consisting of a 17.2 wt% aqueous PEDOT:PSS solution (Clevios PH 1000, Heraeus, Germany), 0.86 wt% dimethyl sulfoxide (DMSO, Sigma-Aldrich, UK), 6.9 wt% fluorosurfactant (Capstone FS-30, Apollo Scientific, UK) and 75 wt% isopropanol (2-Propanol, Sigma-Aldrich, UK).

### Fabrication of the tuneable devices

The expansion-mode device was fabricated as follows. A volume of 25 µL of the PEDOT:PSS compound solution was used to spray on both sides of a 4-times pre-stretched VHB elastomer membrane a 28 mm-wide circular thin layer (estimated thickness lower than 100 nm), to be used as a transparent electrode. Then, the membrane was partially relaxed to a 3.5-times pre-stretch, making the PEDOT:PSS electrode wrinkled, due to the dissimilar stiffness with the elastomer membrane^45^. Then resulting −23.4% area strain corresponded to a reduction of the electrode diameter from 28 to 24.5 mm (Fig. 2a) and an increase of the membrane thickness from 62.5 to 81.6 µm (calculated values). The membrane was finally fixed to a support frame, taking advantage of its adhesive properties.

The contraction-mode device was fabricated in the same way as the expansion-mode one, although without any reduction of the 4-times pre-stretch. So, the membrane thickness was about 62.5 µm and the PEDOT:PSS-coated central area had a diameter of 28 mm. A non-transparent stretchable conductor, consisting of conductive carbon grease (Carbon Conductive Grease 846, M.G. Chemicals, Canada), was smeared on the annular outer region of each side of the membrane, so as to obtain ring-shaped stretchable electrodes. The central circular area was separated by the outer annular area by a gap of 2 mm (Fig. 2b).

The dual-mode device was fabricated combing the procedures described above. So, the membrane thickness was about 81.6 µm and the PEDOT:PSS electrode had a diameter of 24.5 mm. The gap between the circular and annular electrodes was 3.75 mm wide (Fig. 2c).

For all the devices, the PEDOT:PSS layer was oven dried for 30 min at 80 °C. A copper tape was used to connect each electrode to the voltage source.

### Electrical driving

In order to drive the devices, a compact DC-DC high voltage converter (Q-series, EMCO High Voltage Corporation, USA) was used to produce voltages up to 6 kV, which were monitored with a high-voltage probe. Each voltage value was divided by the membrane’s thickness at electrical rest (81.6 or 62.5 µm, as describe above), in order to calculate the applied (nominal) electric field.

### Characterisation of the optical transparency

Transmittance spectra of the tuneable devices were characterised, in the 400–800 nm visible range, using a Perkin Elmer Lambda 950 UV-vis spectrometer with a 10 cm-wide integrating sphere.

### Measurement of the actuation strains

The actuation strains were calculated by processing images (taken at different driving voltages) with the software Image J.

### Microscopic characterisation of the surface wrinkling

The reversible formation of surface wrinkles within the PEDOT:PSS thin films at different area strains was investigated using an atomic force microscope - AFM (NanoWizard 4 BioAFM, JPK Instruments AG, Germany) and a scanning electron microscope - SEM (Inspect F50, FEI, USA). The AFM images were taken with a cantilever probe (NSG01, NT-MDT, Russia) having a resonant frequency of around 150 kHz and a spring constant of 3.5 N/m. The SEM images were taken with an acceleration voltage of 2 kV.

## Supplementary information

Supplementary information

Supplementary Video
