# Supplementary material for: Electrically tuning soft membranes to both a higher and a lower transparency
Source: Sci Rep. 2019 Dec 27;9:20125. doi: 10.1038/s41598-019-56505-9 (PMC6934721; doi:10.1038/s41598-019-56505-9)
Supplement: Supplementary file 1 — Supplementary information [file 41598_2019_56505_MOESM1_ESM.pdf]

Electrically tuning soft membranes to both a higher and a lower transparency

Leihao Chen, Michele Ghilardi, James JC Busfield, Federico Carpi

SUPPLEMENTARY INFORMATION

Video SV1

Supplementary video. Dual-mode device with sequential operation in expansion mode and contraction mode.

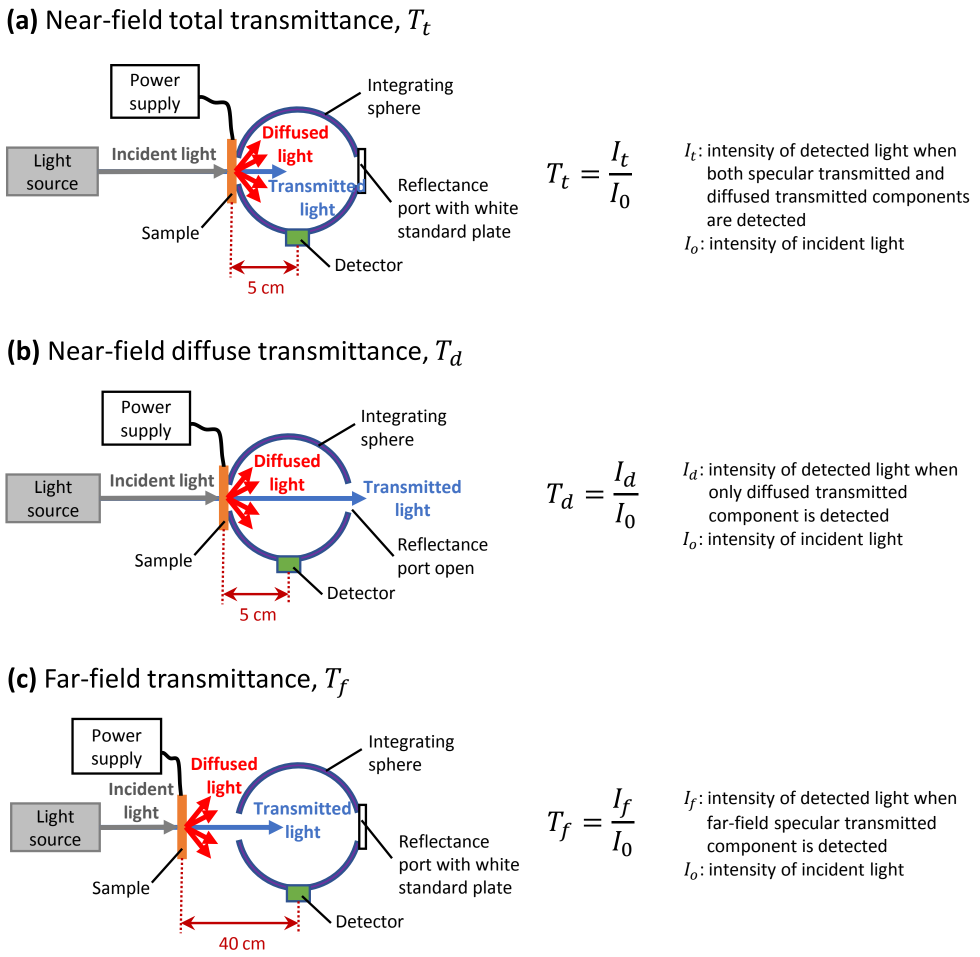

Figure S1. Schematic representation of the UV-Vis spectrometer set-up used to characterise the transmittances: (a) Near-field total transmittance measurement, with both specular transmitted and diffused transmitted light detected; for this test, the transmission port was covered with the sample, whilst the reflectance port was covered with a white standard plate. (b) Near-field diffuse transmittance measurement, with only diffused transmitted light detected; for this test, the transmission port was covered with the sample, whilst the reflectance port was left open. (c) Far-field transmittance measurement, with only specular transmitted light detected; for this test, the transmission port was left open, whilst the reflectance port was covered with a white standard plate.

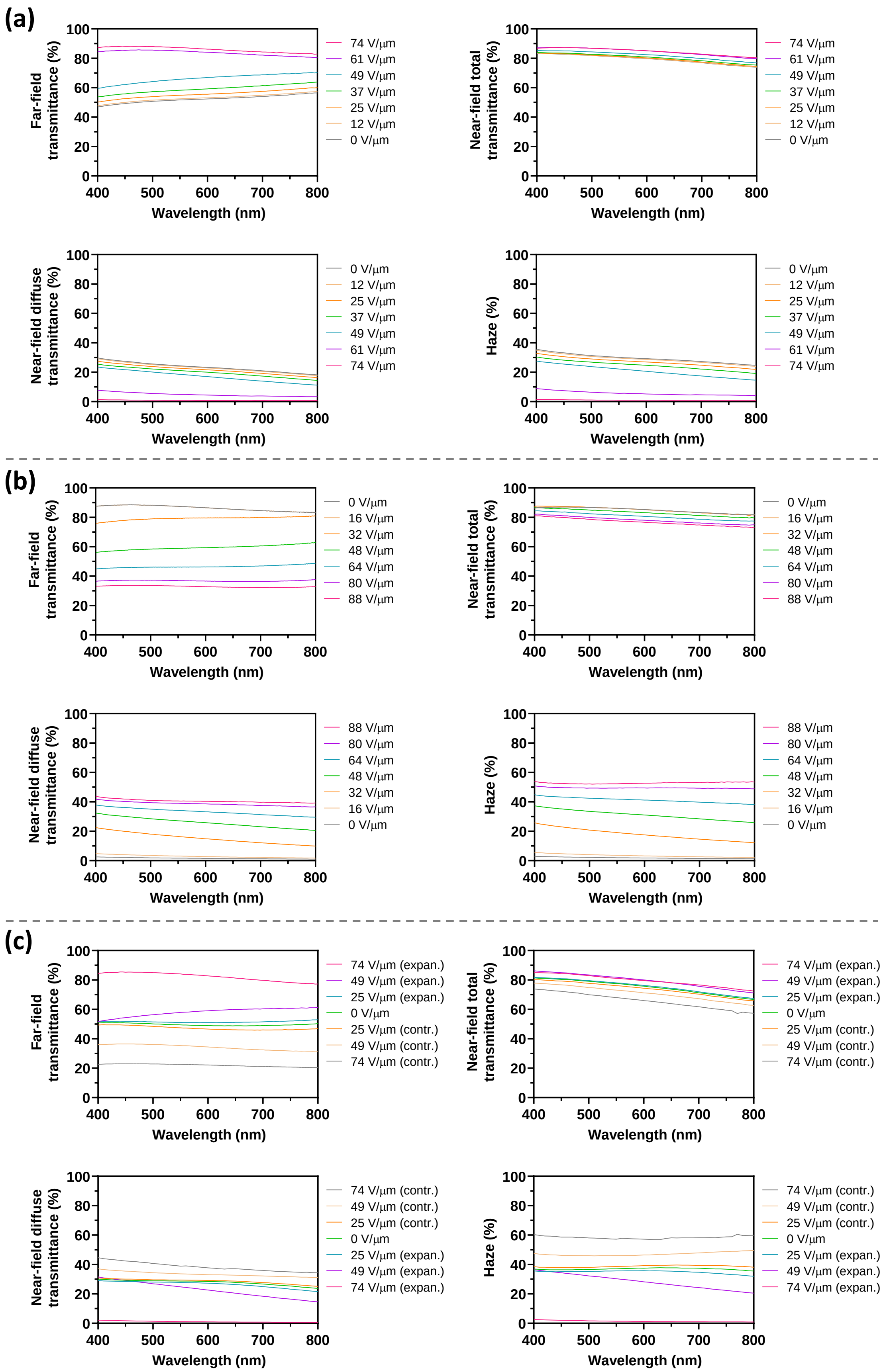

Figure S2. Visible-range spectra of the far-field transmittance, near-field total transmittance, near-field diffuse transmittance and Haze value of the soft membrane-based devices working in (a) expansion mode, (b) contraction mode or (c) dual expansion-contraction mode.

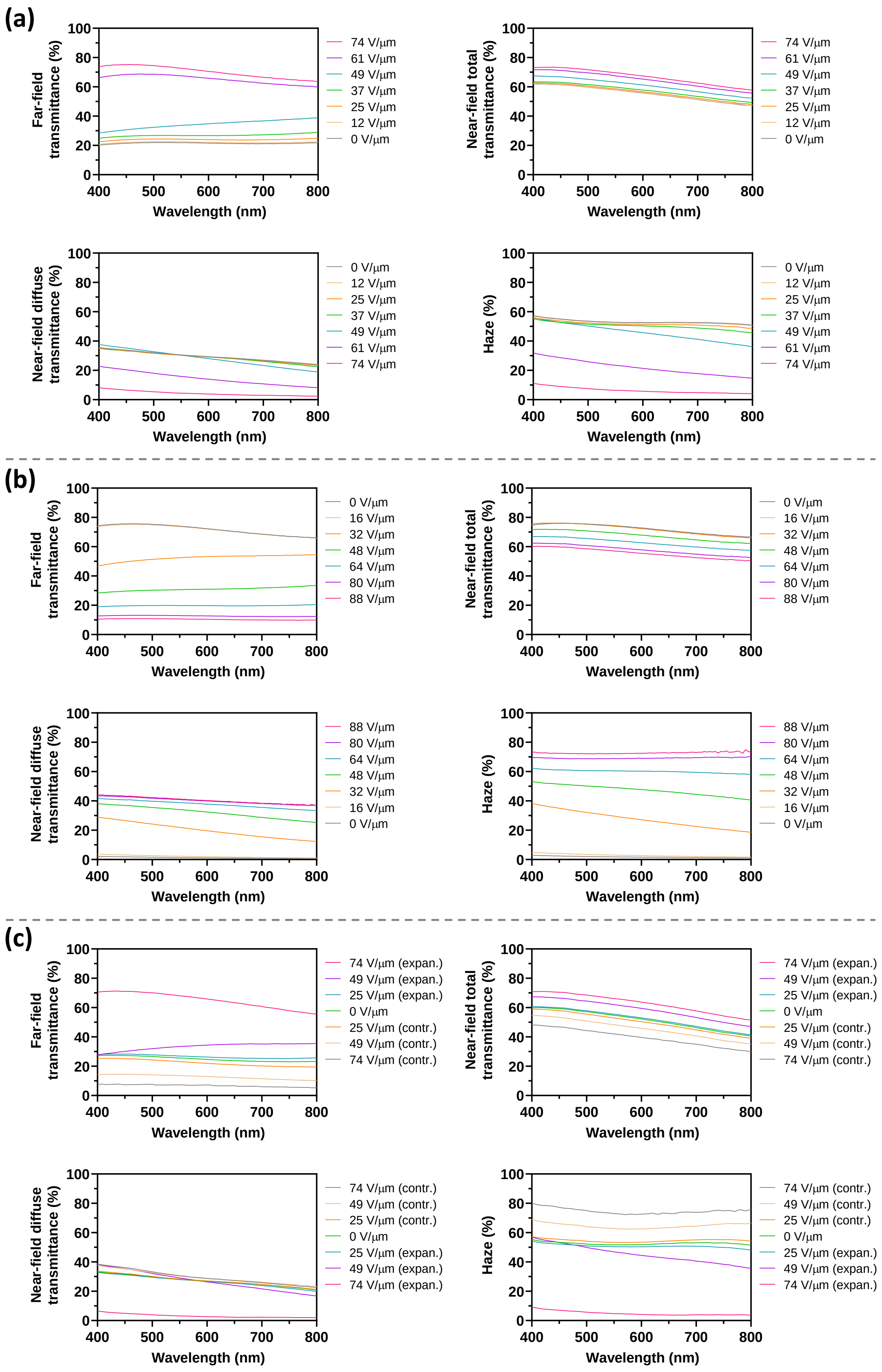

Figure S3. Visible-range spectra of the far-field transmittance, near-field total transmittance, near-field diffuse transmittance and Haze value of two coaxially-aligned devices, both working in (a) expansion mode, (b) contraction mode or (c) dual expansion-contraction mode.

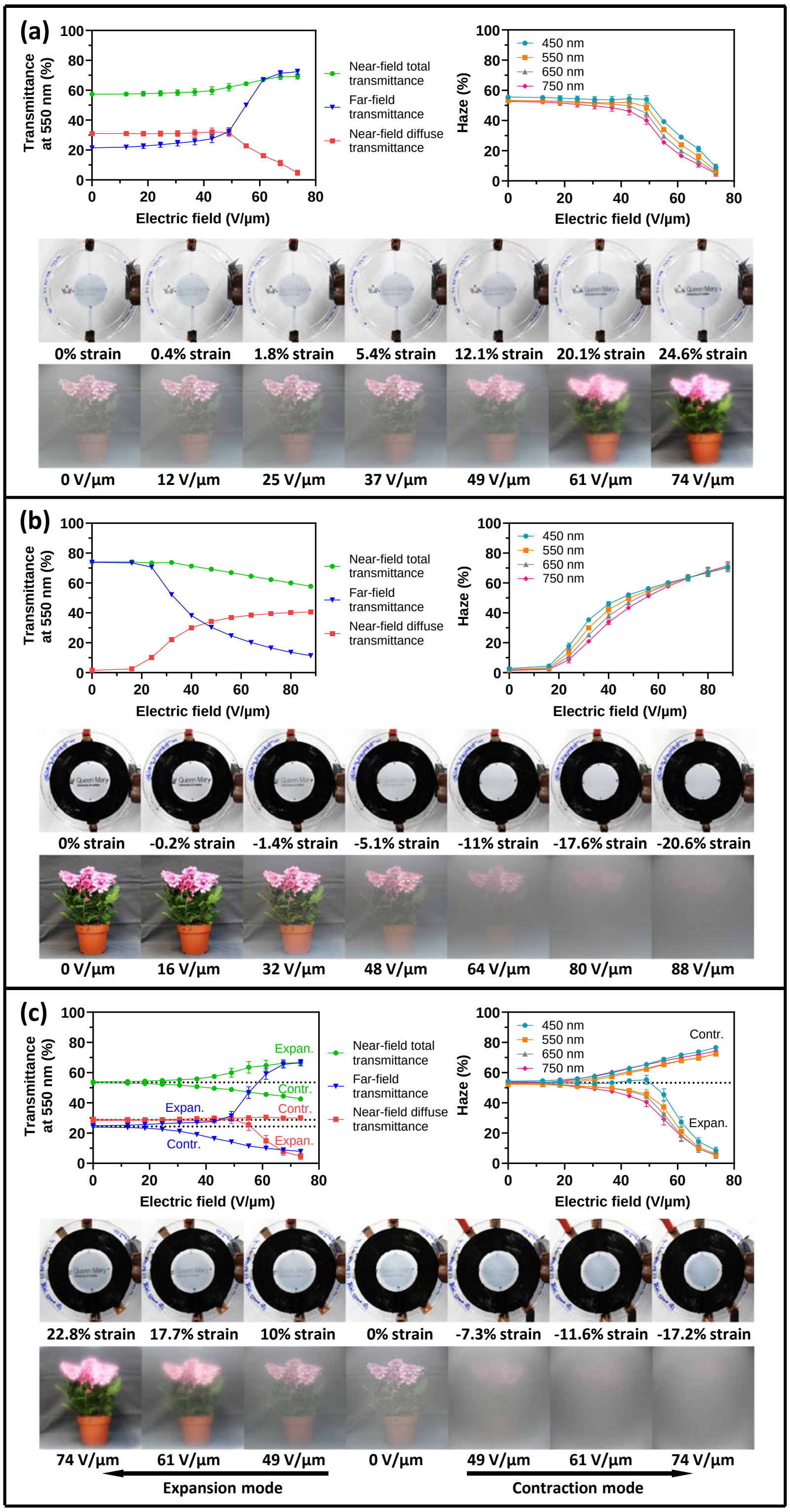

Figure S4. Electro-optical transduction performance of two coaxially-aligned soft membrane-based devices, both working in (a) expansion mode, (b) contraction mode or (c) dual expansion-contraction mode. For each configuration, the electrically-induced variations of the transmittances (near-field total, near-field diffuse and far-field) at 550 nm and the Haze number at different wavelengths are shown. Each data point represents the average value from three sample devices. Error bars corresponding to the standard deviation are included, although most of them are too small to be seen within the 0-100% range of the graph. The photographs visualise the change in transparency due to the specified electric fields, which caused the reported area strains of the PEDOT:PSS window: the first photo set shows the device covering text 3 cm away, whereas the second set shows flowers 100 cm approximately away from a device attached to the camera lens.
